# Response of salivary biomarkers to an empathy triggering film sequence—a pilot study

**DOI:** 10.1038/s41598-021-95337-4

**Published:** 2021-08-04

**Authors:** Christoph Zenzmaier, Jessie Janssen, Christoph Zulmin, Philipp Österreicher, Lea Heinrich, Gerhard Tucek, Susanne Perkhofer

**Affiliations:** 1 Health University of Applied Sciences Tyrol, Innsbruck, Austria; 2 Josef Ressel Centre, Horizons of personalized music therapy—Researching music therapy processes and relationships in selected fields of neurologic rehabilitation, Department of Health Sciences, Institute of Therapeutic Sciences, IMC University of Applied Sciences Krems, Krems an der Donau, Austria

**Keywords:** Biomarkers, Human behaviour

## Abstract

Empathy is a multifaceted phenomenon that is difficult to measure. Self-report questionnaires are the most common and well-validated measures while currently no validated protein biomarkers associated with the empathic reaction have been established. Trigger films have been previously used in psychological research to evoke emotions. Thus, in the present randomized cross-over study we investigated the responses of nine salivary biomarkers that have been related to emotions and stress following an empathy triggering and a control film sequence. Additionally, questionnaires for empathy (Saarbrucken Personality Questionnaire (SPQ)) and current mental stress were applied and participants were asked to assess the film protagonists’ emotions using the Positive and Negative Affect Schedule. Data from 46 participants were included in the analysis. α-Amylase, IgA, IL-1β and estradiol showed a significantly different response between the empathy and control intervention. Moreover, normalized levels of these biomarkers significantly correlated with single scales of the SPQ (control film sequence: α-amylase and IgA with personal distress; estradiol with empathic concern; IL-1β with fantasy; empathy triggering film sequence: IgA with empathic concern, fantasy and the total empathy score). These findings indicated that the observed changes in salivary biomarker levels were reflective of a physiological response to the empathy triggering film sequence. Future studies using different triggers and settings will show if the identified biomarkers can be considered as surrogate markers for empathic reactions in general.

## Introduction

Empathy is a multifaceted phenomenon with varying definitions. The most widely used definitions of the empathy construct are based on affective and cognitive components, whereby cognitive empathy is the ability to understand the thoughts and feelings of others and affective empathy the capability to share the feelings of another person^1^.

Various approaches to measure empathy including self-report, behavioral and neuroscientifc measures have been used and well-validated^2^, with self-report questionnaires being the most common. To date, no validated protein biomarkers associated with the empathic reaction have been established. Thus, in the present study we investigated the response of various biomarkers to an empathy triggering film sequence.

Biomarkers that have been related to emotions, stress or interventions to reduce stress in previous studies were selected. Given that invasive specimen-collection procedures such as blood draws might be stress evoking^3^ and acute stress might affect current empathy^4,5^, only biomarkers detectable in saliva samples were considered to avoid potential confounding. Among these were neuroendocrine markers such as cortisol or testosterone that have been studied in relation to music^6^. Cortisol is a hormone of the hypothalamic-pituitary-adrenocortical (HPA) axis. Dehydroepiandrosterone (DHEA), an antagonist of cortisol, serves in the body as a precursor for the synthesis of other steroid hormones such as androstenedione, testosterone or estradiol, which influence social behavior^7–10^. α-Amylase, a well-characterized marker of the sympathetic nervous system, has been associated with chronic stress, post-traumatic stress disorder or behavior^11,12^. Increasing concentrations of inflammatory markers such as secretory immunoglobulin A (IgA) or interleukin-1 (IL-1) were observed during performing or listening to relaxing or classical music and inflammatory cytokines including IL-1 or IL-8 have also been studied in relation to stress^6,13^.

**Figure 1:**
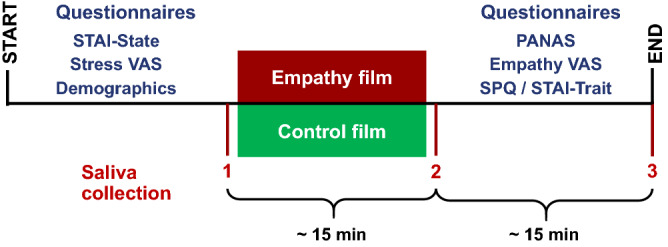
Schematic presentation of data collection sessions. *STAI* State-Trait Anxiety Inventory, *VAS* visual analogue scale, *PANAS* Positive and Negative Affect Schedule, *SPQ* Saarbrucken Personality Questionnaire.

Trigger films have been previously used in psychological research to evoke emotions such as fear, anger, sadness, disgust, amusement, tenderness, excitement, happiness or surprise^14^. In the present study, we investigated the response of nine salivary biomarkers to an empathy triggering compared to a control film sequence. Therefore, biomarker levels prior and after the film interventions were analyzed in comparison to established and well-validated self-report measures for empathy and stress. Data collection was performed as summarized in Fig. 1.

## Results

In total 51 students of the Music Therapy Bachelor and Master degree programs consented to participate in the study and were randomly assigned to one of the two study groups. Data from five participants were excluded from analysis because they did not show up on the second data collection session. The included 46 participants had a median age of 27.5 years (interquartile range 23–36.25) and the vast majority were female (N = 43). Twenty-one students had finished secondary school, 22 had graduated from a university and three received other education.

Participants had a median score of 39 in the State-Trait Anxiety Inventory (STAI)-Trait questionnaire and a total empathy trait score of 47 according to the Saarbrucken Personality Questionnaire (SPQ), with 37 (80%) of them having a total score ≥ 45 thus being considered empathic. Age, trait anxiety and results of the SPQ, including its single dimensions empathic concern (EC), fantasy (FS), personal distress (PD) and perspective taking (PT), are summarized in Table 1.

**Table 1:** Participants’ age, trait anxiety and trait empathy as determined by SPQ (n = 46).

	Median	(IQR)
Age [yrs]	27.5	(23, 36.25)
Trait anxiety	39	(33, 41.25)
**SPQ**		
EC	16	(15, 18)
FS	15	(13.75, 17)
PD	11	(9.75, 12.25)
PT	16	(15, 17)
E	47	(45, 51)

*SPQ* Saarbrucken Personality Questionnaire, *EC* empathic concern, *FS* fantasy, *PD* personal distress, *PT* perspective taking, *E* empathy score (= EC + FS + PT).

### Participants’ assessment of the film protagonists’ emotions

The Positive and Negative Affect Schedule (PANAS) questionnaire was applied as a measure for the participants’ ability to empathize with the protagonists of the film sequences. The perception of film protagonist’s emotions in the empathy film sequence significantly differed from the control film with positive affect (PANAS PA) being significantly lower (median 25 vs. 42, *P* < 0.001) and negative affect (PANAS NA) significantly higher (34 vs. 14.5, *P* < 0.001). Additionally, participants better managed to empathize with the protagonist in the empathy film sequence than with the protagonist in the control film sequence, as determined by self-assessment using a visual analogue scale (empathy VAS; median 84.5 mm vs. 70.5 mm).

Since acute stress potentially interferes with the empathic reaction, baseline levels of STAI-State and a visual analogue stress scale (stress VAS) at the beginning of each data collection session were collected. Acute stress levels at the two data collection sessions did not differ significantly (Table 2).

**Table 2:** Self-reported stress pre, and empathy post empathy trigger or control film sequence.

	Control		Empathy		*P*-value*
	Median	(IQR)	Median	(IQR)	
STAI state	36	(33, 40.25)	36	(32, 43.5)	0.147
Stress VAS [mm]	25	(18.75, 41.5)	30	(18.75, 54.5)	0.061
PANAS PA	42	(37.75, 44)	25	(22, 29)	< 0.001
PANAS NA	14.5	(12, 17)	34	(30, 38.25)	< 0.001
Empathy VAS [mm]	70.5	(59.5, 80.5)	84.5	(75.75, 91.25)	< 0.001

*Wilcoxon signed-rank test.

Several correlations between the psychometric measures were observed. STAI-State and Stress VAS significantly correlated at both data collection sessions (empathy film: correlation coefficient 0.623, *P* < 0.001; control film: 0.559, *P* < 0.001). Empathy VAS after the empathy film significantly correlated with SPQ EC (0.434, *P* = 0.003) and the SPQ total score (0.408, *P* = 0.005).

### Salivary biomarker levels

In order to analyze the biological response to the empathy triggering film sequence, salivary samples for biomarker analysis were collected at three time points (TP), prior the film intervention (TP1), directly after the 10 min film (TP2), and approximately 15 min after the film (TP3). Table 3 summarizes all salivary biomarker levels assayed (α-amylase, androstenedione, cortisol, DHEA, estradiol, IgA, IL-1β, IL-8, and testosterone) pre and post the empathy trigger and the control film sequence. Salivary α-amylase levels were significantly decreased after the control film (TP3 median 178 U/mL vs. TP1 197 U/mL, *P* = 0.023) but did not significantly change after the empathy film (200 U/mL vs. 183 U/mL, *P* = 0.49). In contrast, IgA levels were significantly decreased after the empathy film sequence (TP1 26.5 µg/mL; TP2 21.2 µg/mL, *P* = 0.009; TP3 22.1 µg/mL, *P* = 0.001) but not after the control. Salivary estradiol levels were significantly increased after both film sequences. Significant differences were also observed with both films regarding cortisol and testosterone levels (Table 3).

**Table 3:** Biomarker level pre (time point 1), and post (time points 2 and 3) empathy trigger or control film sequence.

	Intervention	Time point 1		Time point 2		Time point 3	
		Median	(IQR)	Median	(IQR)	Median	(IQR)
α-Amylase	Control	197	(134, 275)	176	(144, 283)	178*	(136, 254)
[U/mL]	Empathy	183	(128, 283)	198	(137, 287)	200	(139, 283)
Androstendione	Control	100	(61, 170)	96	(68, 162)	114	(67, 164)
[pg/mL]	Empathy	107	(61, 141)	110	(66, 156)	114	(51, 171)
Cortisol	Control	2.1	(1.6, 3.3)	1.9**	(1.4, 3.1)	1.7**	(1.3, 3.1)
[ng/mL]	Empathy	1.9	(1.4, 4.1)	2.1*	(1.6, 3.6)	2.3*	(1.6, 3.4)
DHEA	Control	146	(77, 252)	154	(78, 243)	146	(79, 259)
[pg/mL]	Empathy	154	(70, 215)	130	(90, 223)	138	(75, 210)
Estradiol	Control	5.7	(3.0, 8.6)	7.2**	(3.7, 10.8)	6.9*	(3.4, 11.2)
[pg/mL]	Empathy	5.1	(1.8, 7.9)	6.1**	(2.9, 9.9)	6.9**	(3.4, 10.2)
IgA	Control	22.2	(12.6, 30.2)	21.6	(15.0, 32.9)	23.7	(15.4, 34.6)
[µg/mL]	Empathy	26.5	(18.5, 40.2)	21.2**	(13.6, 33.4)	22.1**	(13.3, 33.6)
IL-1β	Control	26.7	(17.6, 70.1)	33.4	(17.2, 67.6)	28.8	(19.0, 63.1)
[pg/mL]	Empathy	33.4	(15.6, 91.6)	26.6	(12.1, 82.3)	25.4	(12.6, 83.3)
IL-8	Control	14.3	(5.3, 148.7)	12.2	(7.1, 191.6)	15.6	(6.7, 107.4)
[pg/mL]	Empathy	20.2	(8.0, 152.5)	17.3	(7.2, 150.6)	15.6	(6.8, 135.0)
Testosterone	Control	45.9	(13.7, 75.3)	42.2	(19.4, 81.2)	47.2**	(18.3, 86.8)
[pg/mL]	Empathy	28.6	(11.9, 72.1)	34.9	(13.4, 76.4)	47.9**	(13.0, 90.7)

Wilcoxon signed-rank Test; *P < 0.05; **P < 0.01 compared with time point 1.

**Figure 2:**
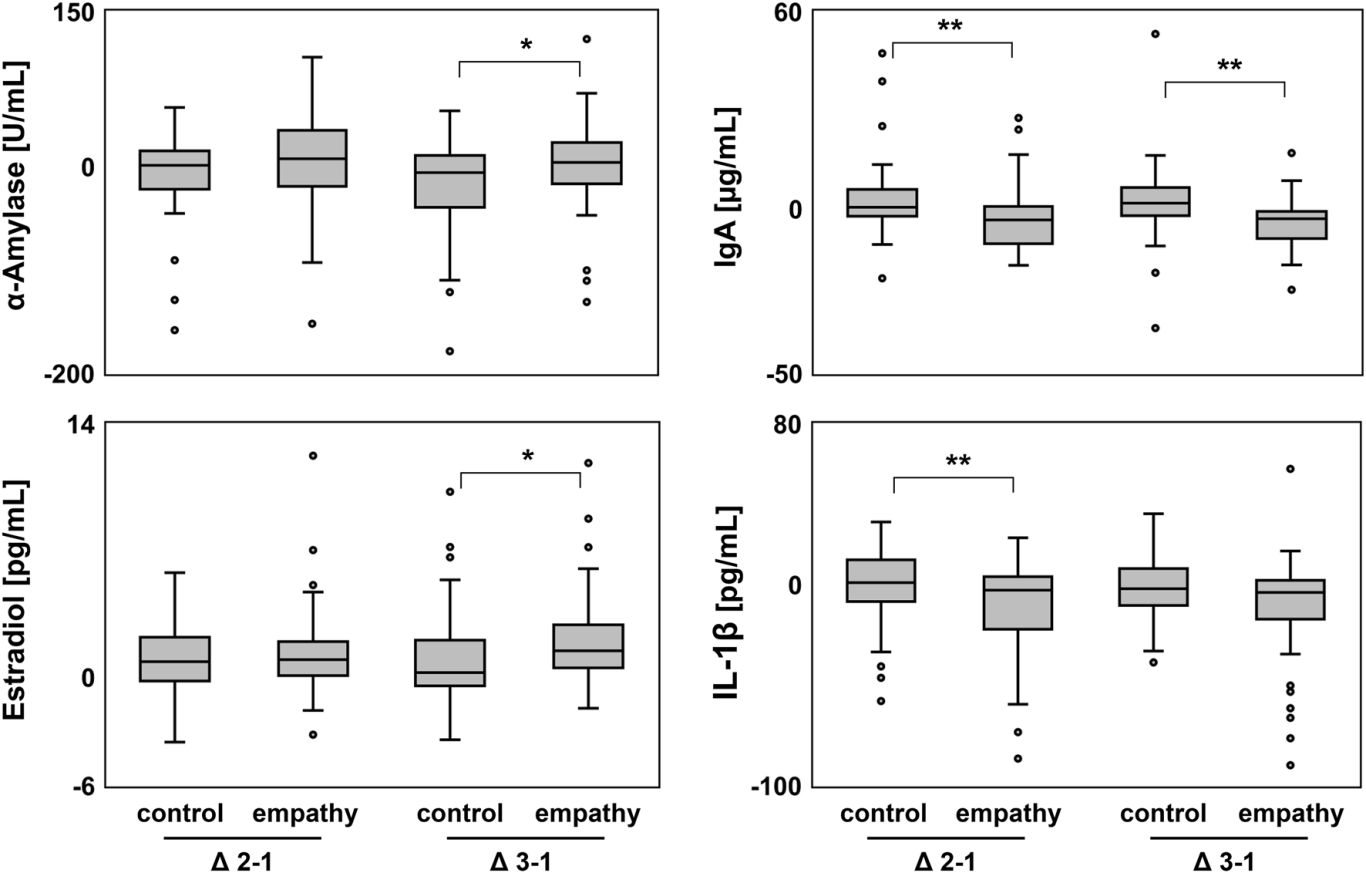
Normalized salivary biomarker levels post empathy trigger or control film sequence. Normalized salivary levels of α-amylase, IgA, estradiol and IL-1β at time point 2 (Δ_2-1_) and time point 2 (Δ_3-1_) post empathy trigger or control film sequence. Statistical significance determined by Wilcoxon signed-rank test. *P < 0.05; **P < 0.01.

To better compare the responses in salivary biomarker levels between the two film interventions, levels after the film sequences were normalized by subtraction of the respective individual baseline levels at TP1. Normalized salivary levels of all biomarkers are summarized in Table 4. Normalized α-amylase levels at TP3 (Δ_3-1_) significantly differed between the film interventions (empathy median 4.1 U/mL vs. control -6.1 U/mL, *P* = 0.042). Normalized IgA levels after the empathy triggering film sequence were significantly lower compared to the control film (Δ_2-1_: − 3.4 µg/ml vs. 0.5 µg/mL, *P* = 0.003; and Δ_3-1_: − 3.0 µg/ml vs. 1.8 µg/mL, *P* < 0.001). Δ_3-1_ estradiol levels were significantly higher (1.5 pg/mL vs. 0.2 pg/mL, *P* = 0.023) and Δ_2-1_ IL-1β significantly decreased (− 2.3 pg/mL vs. 1.0 pg/mL, *P* = 0.004) after the empathy film. Normalized salivary levels of androstendione, cortisol, DHEA, IL-8 and testosterone did not differ significantly between the two film interventions. Biomarkers with significantly different responses to control and empathy film sequences are shown in Fig. 2.

**Table 4:** Biomarker levels post empathy trigger or control film sequence normalized to time point 1.

		Control		Empathy		*P*-value*
		Median	(IQR)	Median	(IQR)	
α-Amylase	Δ_2-1_	1.2	(− 21.1, 15.1)	7.6	(− 19.0, 35.1)	0.105
[U/mL]	Δ_3-1_	− 6.1	(− 39.3, 10.0)	4.1	(− 16.6, 23.3)	0.042
Androstendione	Δ_2-1_	3.3	(− 12.4, 17.2)	0.4	(− 11.3, 15.4)	0.961
[pg/mL]	Δ_3-1_	8.3	(− 13.2, 32.7)	7.3	(− 20.8, 55.0)	0.739
Cortisol	Δ_2-1_	− 0.1	(− 0.6, 0.1)	− 0.2	(− 0.4, 0.2)	0.397
[ng/mL]	Δ_3-1_	− 0.3	(− 0.8, 0.1)	− 0.3	(− 1.0, 0.3)	0.996
DHEA	Δ_2-1_	1.6	(− 18.5, 21.3)	− 0.7	(− 28.0, 16.9)	0.623
[pg/mL]	Δ_3-1_	3.8	(− 22.1, 57.1)	− 1.8	(− 31.2, 25.3)	0.050
Estradiol	Δ_2-1_	0.9	(− 0.2, 2.2)	1.0	(0.1, 2.0)	0.495
[pg/mL]	Δ_3-1_	0.2	(− 0.5, 2.0)	1.5	(0.5, 2.9)	0.023
IgA	Δ_2-1_	0.5	(− 2.2, 5.9)	− 3.4	(− 10.6, 0.7)	0.003
[µg/mL]	Δ_3-1_	1.8	(− 2.0, 6.5)	− 3.0	(− 9, − 0.8)	< 0.001
IL-1β	Δ_2-1_	1.0	(− 8.7, 11.9)	− 2.3	(− 20.7, 4.0)	0.004
[pg/mL]	Δ_3-1_	− 1.9	(− 8.7, 8.0)	− 3.9	(− 16.1, 5.1)	0.117
IL-8	Δ_2-1_	0.0	(− 2.8, 5.7)	1.6	(− 6.2, 10.7)	0.882
[pg/mL]	Δ_3-1_	− 0.5	(− 4.5, 7.0)	− 1.6	(− 15.1, 3.2)	0.134
Testosterone	Δ_2-1_	2.7	(− 1.6, 7.4)	1.5	(− 1.2, 8.1)	0.573
[pg/mL]	Δ_3-1_	3.9	(− 0.2, 13.1)	4.0	(− 0.5, 13.9)	0.851

Δ_2-1_…normalized biomarker level at time point 2 (level time point 2 – level time point 1); Δ_3-1_…normalized biomarker level at time point 3 (level time point 3 – level time point 1); *Wilcoxon signed-rank test.

### Correlation between salivary biomarker levels and trait empathy

Finally, correlations between salivary biomarkers that significantly responded to the empathy triggering film sequence and the surveyed psychometric measures were analysed using spearman’s rho. Normalized levels of all four biomarkers were correlated with single dimensions of the SPQ at a significance level of < 0.01 (Table 5). Changes in α-amylase and IgA levels in the control, but not the empathy intervention, correlated with PD, Δ_2-1_ IL-1β levels with FS, and Δ_3-1_ estradiol levels with EC. Δ_3-1_ IgA levels after the empathy film sequence significantly correlated with the dimension EC and FS and with the SPQ total empathy trait score. No significant correlations between salivary biomarkers and PANAS or the empathy VAS were observed.

**Table 5:** Spearman correlation between trait empathy as determined by SPQ and normalized salivary biomarker levels.

		α-Amylase				Estradiol				IgA				IL-1β			
		Control		Empathy		Control		Empathy		Control		Empathy		Control		Empathy	
		Δ_2-1_	Δ_3-1_	Δ_2-1_	Δ_3-1_	Δ_2-1_	Δ_3-1_	Δ_2-1_	Δ_3-1_	Δ_2-1_	Δ_3-1_	Δ_2-1_	Δ_3-1_	Δ_2-1_	Δ_3-1_	Δ_2-1_	Δ_3-1_
EC	CC	.278	.204	.084	.208	− .196	.430**	.001	− .067	− .127	− .162	− .070	.433**	− .147	.122	− .047	− .102
	*Sig*	*.062*	*.175*	*.585*	*.170*	*.191*	***.003***	*.994*	*.659*	*.399*	*.282*	*.643*	***.003***	*.341*	*.423*	*.764*	*.516*
FS	CC	.210	.005	.050	.318*	− .178	− .276	− .158	.319*	.010	− .002	− .133	.449**	.425**	− .187	− .185	.302*
	*Sig*	*.162*	*.972*	*.746*	*.034*	*.237*	*.063*	*.294*	*.030*	*.945*	*.989*	*.378*	***.002***	***.004***	*.219*	*.235*	*.049*
PD	CC	.377**	.491**	− .212	− .063	− .053	− .137	− .014	− .231	.454**	.420**	− .128	− .104	.140	.165	− .203	− .175
	*Sig*	***.010***	***.001***	*.162*	*.681*	*.724*	*.364*	*.926*	*.122*	***.002***	***.004***	*.395*	*.491*	*.366*	*.279*	*.193*	*.261*
PT	CC	.122	.080	.075	.151	.312*	.043	− .118	− .176	.142	.082	.075	− .075	.016	.043	.219	.247
	*Sig*	*.419*	*.599*	*.624*	*.322*	*.035*	*.775*	*.434*	*.242*	*.346*	*.589*	*.621*	*.620*	*.917*	*.781*	*.159*	*.111*
E	CC	.291	.089	.149	.376*	− .019	− .278	− .110	− .246	− .027	− .079	− .007	.380**	− .221	− .016	.026	− .018
	*Sig*	*.050*	*.557*	*.328*	*.011*	*.900*	*.061*	*.468*	*.099*	*.859*	*.603*	*.961*	***.009***	*.149*	*.915*	*.871*	*.910*

*EC* empathic concern, *FS* fantasy, *PD* personal distress, *PT* perspective taking, *E* empathy score (= EC + FS + PT), *CC* correlation coefficient, *Sig* significance; *significance level 0.05; **significance level 0.01. CCs with *P* < 0.01 were highlighted in bold.

## Discussion

To our knowledge, this is the first study that investigated the effect of an empathy triggering film sequence compared with a control intervention on salivary biomarker levels. We thereby observed a significant difference in salivary α-amylase levels that were mainly based on decreased α-amylase after the control but not the empathy triggering intervention. Salivary α-amylase is a well-accepted marker of the sympathetic nervous system that increases in response to psychological stress^11,15^. Thus, our findings might be reflective of a stress reduction during the control compared to the emotionally charging empathy intervention.

Consistently, differences in salivary levels of the inflammatory marker secretory IgA were observed. IgA is also frequently used as a surrogate salivary marker for stress, and we and others showed a negative correlation between the IgA and mental stress^15,16^. Therefore, the decrease in IgA levels after the empathy triggering film could at least in part result from perceived psychological stress caused by the film intervention.

In agreement with their use as surrogate markers for stress, normalized α-amylase and IgA levels after the control film correlated with the dimension of personal distress (PD) of the SPQ. Similarly to IgA, levels of a second inflammatory marker, IL-1β were lower after the empathy triggering film sequence. Salivary levels of the pro-inflammatory cytokine IL-1β were found to increase in response to acute stress in several studies^17^. Inflammatory cytokines, in particular IL-1β, have also been linked to aggressive behaviors such as hostility, anger and irritability in animal studies and humans^18,19^. Moreover, in a study investigating the relationship between emotional states and inflammatory cytokines in response to acute stress induced by an academic exam, IL-1β plasma and saliva levels were positively associated to the anger state but not STAI-State^20^. Unlike α-amylase and IgA, normalized IL-1β levels after the control film in our study did not correlate with PD but with the dimension fantasy (FS). To analyse the potential association with anger state, in future research anger state could be assessed by questionnaires pre and post empathy triggering interventions.

The levels of the sex steroid hormone estradiol were correlated with the empathic concern (EC) scale after the control film. EC is defined as “sympathy and concern for others in unfortunate situations” and thus refers to affective empathy^21^. Estradiol administration resulted in increased emotional reactivity in males in a double-blind placebo controlled study^22^. Consistently, hormonal status modulated affective empathy in women^23^. The use of oral contraceptives, and thus suppressed production of estradiol and progesterone has additionally been associated with decreased cognitive empathy^24,25^.

Whereas the normalized levels of α-amylase and IgA in the control intervention correlated with PD, biomarker responses to the empathy triggering film sequences were not related to distress trait. However, IgA levels were correlated to EC, FS and the total empathy score of the SPQ. FS is defined as “the ability of respondents to transpose themselves into the feelings of fictional characters in books, movies, etc.”^21^ and is thus rather reflective of cognitive empathy. Taken together, the observed findings from salivary biomarker analysis and their correlation with the SPQ indicate a physiological response to the empathy triggering film sequence different from a stress reaction.

Using a similar setting, decreased levels of testosterone were recently reported in response to an empathy inducing movie sequence^26^. The findings of this study are not directly comparable, as no control intervention was used. However, in the present study we observed increased testosterone levels after both film sequences. The reasons for these conflicting findings are presently unknown, but might be due to differences in experimental settings, such as different movie sequences used, different study populations, saliva collection time points and methods, and ELISA kits used. Testosterone administration has been demonstrated to impair cognitive empathy-related performance in women but not in men^27,28^. Thus, potential gender differences in the role of sex steroid hormones on empathy have to be considered when interpreting and comparing study findings.

The observed significant differences in PANAS and the empathy VAS indicate the suitability of the selected film sequence to trigger empathy. To exclude bias by different individual perceived baseline stress levels between the data collection sessions, participants were randomly assigned to the two intervention groups. Additionally, perceived stress levels at both data collection units were assessed using STAI-State and stress VAS. As expected, pre intervention stress levels did not differ between the two film sequences. The observed correlation between STAI-State and the stress VAS is in line with our previous findings^16,29^.

The present study has several limitations that need to be considered when interpreting its findings. The overall number of participants was limited. Moreover, the group of participants was rather homogenous which may affect the generalizability of our findings. All participants were students of music therapy bachelor or master programs. Thus, the participants had a similar educational and social background, were from the same age group and the vast majority were female. Future research has to demonstrate the validity of our findings in other/representative samples.

Although settings in the two data collection sessions were comparable regarding factors such as location of data assessment or film sequence duration, there were differences in some criteria for emotion eliciting films (defined by Rottenberg et al.^30^). The films differed e.g. in number of actors, sex of the main protagonist, color, picture motion, or complexity.

For the assessement of empathy, the PANAS questionnaire used in this study was modified to, instead of a self-report measure, estimate emotions of the film protagonists. As the modified version was not validated, findings need to be interpreted cautiously.

Given the discussed associations of the identified biomarkers with psychological stress, future studies should assess stress levels pre and post empathy triggering interventions to better differentiate between effects of empathic and stress responses for example by using the stress VAS. The emotions mediated by the used empathy triggering film sequence were clearly negative ones, as demonstrated by the increased PANAS NA and decreased PA scores. Thus, the response in salivary biomarkers observed in the present study is only reflective of an empathic response to triggers with similar emotional quality. To evaluate if the identified biomarkers can be considered as surrogate markers for empathic reactions in general, future studies using different triggers and settings should be conducted. These studies should assess salivary levels of α-amylase, IgA, IL-1β and estradiol. Given the observed gender differences in the role of sex steroid hormones on empathy, we recommend to additionally include testosterone as a potential biomarker as it might play a greater role in male participants.

Empathy in the course of caregiver and patient encounters improves patients’ satisfaction and also contributes to patient outcomes^21,31^. Empathy allows health professionals, and allied health professionals, such as music therapists, to react to the experiences and concerns of their patients, which is essential to establish a positive relationship between the therapist and the patient^31^. Thus, it is important that students and allied health professionals be trained in empathy, and established biomarkers combined with a standardized trigger for empathy could be used to evaluate the effectiveness of such trainings.

## Methods

### Design

A cross-over design was applied in which each participant was randomly assigned to one of two experimental conditions, empathy trigger film sequence versus control film sequence for the first data collection session. For the second data collection session, each participant was then assigned to the respective other experimental condition.

### Recruitment

Students of the music therapy bachelor and master degree programs at a local university of applied sciences were invited to participate in the study. An information sheet was sent out a week before the first scheduled data collection day. In addition, aims, procedure and voluntary nature of the study were explained via an informal presentation by the course leaders, were the students could ask any remaining questions. Students were included in the study if they were aged between 18 and 99 years, and provided written informed consent.

### Psychometric measures

Psychological stress was assessed using the German Version of the well-validated Spielberger STAI, which is the most commonly used self-report to measure current anxiety. The STAI consists of two separate scales, Trait Anxiety Inventory (STAI-Trait) and the State Anxiety Inventory (STAI-State). The Trait Anxiety Inventory measures the general tendency to fear, whereas the State Anxiety Inventory measures the current anxiety. Each scale consists of 20 questions, ranging from one to four^32,33^.

Higher scores (range 20–80) indicate greater levels of anxiety and to define probable clinical levels of anxiety a cutoff score of 40 is commonly used.

Additionally, a previously described stress VAS was used for self- assessment of acute stress^16,29^. The scale consisted of a horizontal line of 100 mm length ranging from no stress to biggest stress which asks: “how stressed do you feel at this moment?”. Six emoticons are displayed to visually represent the different stress levels.

As a self-report measure for trait empathy the SPQ^34^ was used. The SPQ is the German version of the interpersonal reactivity index (IRI)^35,36^ and provides measures of dispositional tendencies in the four areas empathic concern (EC), fantasy (FS), personal distress (PD) and perspective taking (PT), each assessed as a 4-item scale with values ranging from 4–20. Additionally, a total empathy score (E = EC + FS + PT) was calculated as suggested previously^37,38^. Higher scores of the total score (range 12–60) indicate greater levels of empathy and a cut-off value of 45 has been suggested to classify persons as empathic^38^.

The PANAS is a self-report questionnaire that consists of 20 items rated from one (not at all) to five (very much). It comprises two 10-item scales to measure both positive and negative affect^39^. For the purpose of the present study, instead of self-reporting, participants were asked to rate the emotions of the protagonists in the film sequences.

Additionally, a visual analogue scale was used for self-assessment of current empathy (empathy VAS). The scale was used to answer the question “How did you manage to empathize with the person?” and consisted of a horizontal line of 100 mm length ranging from “I did not manage” to “I managed very well”.

### Determination of salivary biomarker levels

Salivary samples were collected using Salivette Cortisol (Sarstedt) sampling devices and stored at − 20 °C until assayed. Samples were thawed and subsequently centrifuged for 10 min at 3000 rcf. Concentrations of salivary analytes were determined in duplicates using commercially available enzymatic assays for α-amylase (Saliva Assay RE80111), androstenedione (Saliva ELISA RE52671), cortisol (Saliva ELISA RE52611), DHEA (Saliva ELISA RE52651), 17beta-estradiol (Saliva ELISA RE52601) and IgA (Saliva ELISA DM59171; all Tecan/IBL International); IL-1 beta (Uncoated ELISA 88–7261) and IL-8 (Uncoated ELISA 88–8086; both Thermo Fisher Scientific); and testosterone (ELISA Kit 402510; Neogene Corporation) according to manufacturers’ instructions.

### Film

As a trigger film for an empathic response the freely available video "ReMoved- Torn Out" was used (https://www.youtube.com/watch?v=lOeQUwdAjE0). With this video the filmmakers intend to create awareness, to encourage and to support the training of foster parents. It is about a 10-year-old girl that has been removed from her home and separated from her younger brother and now tries to find her way through the foster care system. The first 10:02 min of the movie have been used for the present study due to the change in the quality of the transmitted emotion towards the end of the film. Permission to use this film for this study was obtained (personal communication). A control film sequence with identical length (10:02 min) was specifically generated for this study. It displayed an actor talking about his holiday in the mountains. It was anticipated that this video evokes little emotional quality.

### Data collection and procedure

The data collection consisted of two data collection session that took place on two days within a month. After the participants provided informed consent, they were randomly split into two groups and each group was led to a different room in order to allow the study to run in parallel. The participants were blinded for group allocation (empathy trigger film sequence or control film sequence).

To survey the current baseline stress-level at the day of data collection, the participants completed the STAI-State and the stress VAS. Additionally, age, gender and education were surveyed by a demographic questionnaire. Subsequently, saliva samples were collected using Salivette Cortisol (Sarstedt) sampling devices (saliva time point 1). Then the respective film sequences were shown to each group (either the empathy trigger film sequence of the control film sequence), and directly thereafter, again saliva samples were collected (saliva time point 2).

In the last phase of the data collection unit the test persons were asked to assess the emotions of the protagonist of the video sequences with regard to their quality and intensity using the PANAS. Moreover, participants filled out the empathy VAS. In addition, the SPQ was completed as a measure for the participants’ trait empathy. Finally, the participants collected saliva samples for the third time (saliva time point 3).

The second data collection session was carried out identically, with the difference that each group was shown the other film sequence. In addition, instead of the SPQ, trait anxiety was surveyed in the last phase of the second data collection session. Figure 1 depicts the time course of the first and second data collection units, which lasted about 45 min.

### Analysis

All statistical analyses were performed using IBM SPSS Statistics Version 24.0 (IBM Corp. https://www.ibm.com/products/spss-statistics). Salivary biomarker levels were transformed into relative variables by subtracting the baseline values from the first saliva collection of the respective data collection session (TP1). The calculated relative values were thus referred to as ∆ 2–1 (for TP2) and ∆ 3–1 (for TP3).

In order to test for normal distribution, the Lilliefors-corrected Kolmogorov–Smirnov test was used. As the majority of parameters were not distributed normally, data are presented as median and interquartile range (IQR).

Data of participants who did not show up for both data collection session were excluded from the analysis (listwise deletion). Other missing data were scarce. The Wilcoxon signed-rank test was used to evaluate differences between the two data collection sessions, whereby missing data were handled by pairwise deletion (available-case analysis). *P* values < 0.05 were considered statistically significant.

The spearman correlation was used to explore correlations between individual parameters whereby correlations with *P* < 0.01 were considered statistically significant, and correlations with a coefficient > 0.1 as weak, > 0.3 as moderate and > 0.5 as strong.

### Ethical considerations

The study was conducted according to the principles of the Declaration of Helsinki. Students received oral and written information, and all participants signed an informed consent. Participation was voluntary and participants could withdraw from the study at any time without any consequences. Confidentiality was assured by pseudonymization of data. Participants did not receive any financial incentives. The study protocol was sent to the ethics committee in Lower Austria (NÖ Ethikkommission, St. Pölten, Austria), which confirmed no further ethics application was necessary. IMC University of Applied Sciences Krems approved all study protocols.
